# Diagnosis of Peripheral Lung Lesions via Conventional Flexible Bronchoscopy with Multiplanar CT Planning

**DOI:** 10.1155/2016/5048961

**Published:** 2016-11-13

**Authors:** Marianne Anastasia De Roza, Kien Hong Quah, Cheong Kiat Tay, Weiquan Toh, HuiHua Li, Ganesh Kalyanasundaram, Devanand Anantham

**Affiliations:** ^1^Department of Respiratory and Critical Care Medicine, Singapore General Hospital, Singapore; ^2^Division of Research, Singapore General Hospital, Singapore

## Abstract

*Background.* Conventional flexible bronchoscopy has limited sensitivity in the diagnosis of peripheral lung lesions and is dependent on lesion size. However, advancement of CT imaging offers multiplanar reconstruction facilitating enhanced preprocedure planning. This study aims to report efficacy and safety while considering the impact of patient selection and multiplanar CT planning.* Method.* Prospective case series of patients with peripheral lung lesions suspected of having lung cancer who underwent flexible bronchoscopy (forceps biopsy and lavage). Endobronchial lesions were excluded. Patients with negative results underwent CT-guided transthoracic needle aspiration, surgical biopsy, or clinical-radiological surveillance to establish the final diagnosis.* Results.* 226 patients were analysed. The diagnostic yield of bronchoscopy was 80.1% (181/226) with a sensitivity of 84.2% and specificity of 100%. In patients with a positive CT-Bronchus sign, the diagnostic yield was 82.4% compared to 72.8% with negative CT-Bronchus sign (*p* = 0.116). Diagnostic yield was 84.9% in lesions > 20 mm and 63.0% in lesions ≤ 20 mm (*p* = 0.001). Six (2.7%) patients had transient hypoxia and 2 (0.9%) had pneumothorax. There were no serious adverse events.* Conclusion.* Flexible bronchoscopy with appropriate patient selection and preprocedure planning is more efficacious in obtaining a diagnosis in peripheral lung lesions compared to historical data. This trial is registered with ClinicalTrials.gov Identifier: NCT01374542.

## 1. Introduction

The diagnosis of peripheral lung lesions in patients who are suspected of having lung cancer remains a challenge. The overall diagnostic sensitivity of conventional bronchoscopic lung biopsy in these patients is reported at approximately 57% [1]. The diagnostic yield is affected by the combined use of sampling techniques such as forceps, washing, and needle aspiration, as well as number of samples obtained, size of the lesion, and the presence of computed tomography- (CT-) Bronchus sign that is the presence of an airway leading directly into the pulmonary lesion [2, 3].

An alternative diagnostic modality is CT-guided transthoracic needle aspiration/biopsy that has a pooled diagnostic sensitivity of approximately 90% [4]. The complications associated with this procedure include a high pneumothorax rate of 15% (chest tube insertion rate of 6.6%). Pulmonary hemorrhage rate is less common at 1% but up to 18% of these patients required blood transfusion. Air embolism, tumor seeding, and chest wall hematoma have also been reported [5]. Furthermore, in many institutions, CT-guided transthoracic needle aspiration can only be performed in an inpatient setting. These limitations have spurred the development of navigational bronchoscopy technology to improve the diagnostic yield of a bronchoscopic approach while retaining its benefits that is relatively low complication rate and performed in an outpatient setting. These technologies include radial endobronchial ultrasound (EBUS), virtual navigation bronchoscopy, and electromagnetic navigation. Despite showing some promise, the diagnostic yield of these techniques have plateaued at 67–73% [6]. There is also an associated monetary cost to these advanced techniques and a learning curve for the endoscopists.

The data on bronchoscopic sampling for suspected peripheral bronchogenic carcinoma is also dated with only 4 of 34 studies published after the year 2000 [4]. In current practice, preprocedure planning on an electronic image management system with CT multiplanar reconstruction is increasingly common and can provide some of the benefits associated with virtual bronchoscopy navigation and electromagnetic navigation. In addition, CT resolution has improved with 1.5 to 3 mm slice thickness readily available. The impact of these improvements on bronchoscopy has not been evaluated. The aim of this study was to report “real-world” data on the diagnostic yield and safety of conventional flexible bronchoscopy with appropriate patient selection combined with planning on multiplanar CT in the evaluation of suspected peripheral lung cancer.

## 2. Method

A prospective database of all patients undergoing flexible bronchoscopy at the Singapore General Hospital Endoscopy Centre is maintained and data over 30 months (June 2011–December 2013) was analyzed. This was after multiplanar reconstruction and fine slice thoracic CT thorax became the norm in the institution. Institutional review board approval (number 2011/350/C) was obtained and ClinicalTrials.gov identifier is NCT01374542. This database streams data from patient's endoscopy reports to ensure completeness of data collection. Post hoc chart review was used to establish the final diagnosis.

Inclusion criteria for this study were as follows: (1) all patients that had a prior CT thorax performed with radiological features consistent with malignancy as reported by dedicated body CT radiologists; (2) CT thorax with thickness of reconstructed image sections set at 1.5 to 3 mm and multiplanar reconstruction in the coronal and transverse planes that could be viewed and manipulated on an electronic image management system available; and (3) clinical history suspicious for malignancy such as history of smoking, presence of hemoptysis, or weight loss. Patients who had any evidence of endobronchial lesion on endoscopy were excluded. Patients who had mediastinal adenopathy or peribronchial lesions had endobronchial ultrasound guided transbronchial needle aspiration and were also excluded. Additionally, patients who were lost to follow-up or declined further imaging or investigations were excluded from analysis.

Conventional flexible bronchoscopy was performed by either attending pulmonologists (10 doctors) or pulmonary fellows (6 doctors) under supervision using a standard therapeutic video bronchoscope. This had a 6.0 mm diameter and 2.8 mm working channel. All cases were performed under moderate sedation with fentanyl and midazolam, as well as topical 2% lidocaine. Transbronchial forceps (Olympus Endojaw FB-211D alligator cup forceps; Olympus, Tokyo, Japan) biopsy was performed for all cases in the targeted bronchopulmonary segment. The standard institutional practice is to obtain 6 biopsy specimens. However, bleeding and patient's inability to continue to tolerate bronchoscopy prevented this standard from being achieved in some cases. Bronchoalveolar lavage (BAL) or bronchial washing were also performed at the same sitting prior to the forceps biopsies. Brushing and peripheral transbronchial needle aspiration were not performed for the purpose of standardization. Portable C-arm fluoroscopy was utilized in a single plane but no additional navigational tools were used.

In the data analysis, lesions were classified as >20 mm or ≤ 20 mm based on the largest dimensions of the pulmonary lesions on multiplanar CT scans. CT-Bronchus sign was designated as positive if an airway was identified leading into the target lesion. This would correspond to the 2 Tsuboi variations that are associated with an improved diagnostic yield: (1) bronchus patent up to the tumor and then cut off and (2) bronchus penetrating and contained within the tumor [7]. Patients with bronchus compressed by the tumor with intact mucosa or bronchus constricted by perimucosal tumor spread causing either smooth or irregular narrowing were classified as CT-Bronchus sign negative.

The final diagnosis was based on histology and culture results. A positive result for peripheral lung cancer was made if histology from tissue biopsy and/or BAL cytology showed the presence of malignancy. Bronchoscopic yield was still a success if a confirmed diagnosis other than malignancy could be made, such as infection that was compatible with the clinical and radiological picture. Patients with nondiagnostic results either underwent further tissue sampling with alternative modalities or were followed up with radiological surveillance. Further tissue sampling was performed by CT-guided transthoracic needle aspiration or surgical biopsy. Those on radiological surveillance were followed up for 24 months for stability or progression of the lesion. Benign lesions on CT showed either resolution or stability for at least 24 months.

In the statistical analysis of the data, categorical data was reported in terms of frequency and proportion, while continuous data was reported in terms of mean (standard deviation) or median (range) where applicable. Logistic regression was carried out to evaluate the effect of potential factors affecting bronchoscopy success and complication rate. Receiver Operating Characteristic (ROC) curve was performed to evaluate the performance of diagnosis of cancer using bronchoscopy. All analyses were done using R 3.1.3 (https://www.r-project.org/) with significance level at 0.05.

## 3. Results

A total of 229 patients met inclusion criteria. Of these, 3 patients were excluded as they were lost to follow-up and diagnoses could not be determined. Therefore, 226 patients were included in the analysis. 61.5% were male and median age was 64 years (range 30 to 90). The prevalence of malignancy was 78.7% (178/226). Mean lesion size was 40 ± 21 mm and 179 (79.5%) of the lesions were >20 mm. The CT-Bronchus sign was identified in 165 (73.7%) patients. Median bronchoscopy duration was 25 minutes (range 5 to 75). The median midazolam dose was 2.5 mg (range 0 to 12.0) and fentanyl dose was 50.0 Mcg (range 0 to 100.0). Median number of forceps biopsies taken was 8 (range 2 to 14). 86.7% of patients had a minimum of 6 biopsies or more (Table 1).

A confirmed final diagnosis was available in 215 patients (95.1%). 181 were successfully diagnosed from bronchoscopic specimens. Among patients with a positive bronchoscopy diagnosis, 84.5% were diagnosed with cancer and 15.5% were diagnosed with infections. Adenocarcinoma was the most common subtype of cancer (Table 2). Out of the 215 cases, there were 34 false negatives. All 34 patients underwent repeat tissue biopsy using other means such as CT-guided transthoracic or surgical biopsy. 25 out of 34 cases were diagnosed with malignancy and non-small-cell lung cancer (22 cases) was the most common. The other 9 out of 34 false negatives had nonmalignant disease that is pulmonary infections or anthracosis (Figure 2).

No confirmed final histological or microbiological diagnosis was available in 11 cases (11/226, 4.86%) for which bronchoscopic biopsy revealed either necrotic or inflammatory yield. These 11 patients had repeat imaging using CXR or CT thorax: 8 showed complete resolution and 3 were stable over 24 months. These 11 cases were classified as true negatives and the clinicoradiological pattern was consistent with a benign cause such as postinflammatory changes.

**Figure 1:**
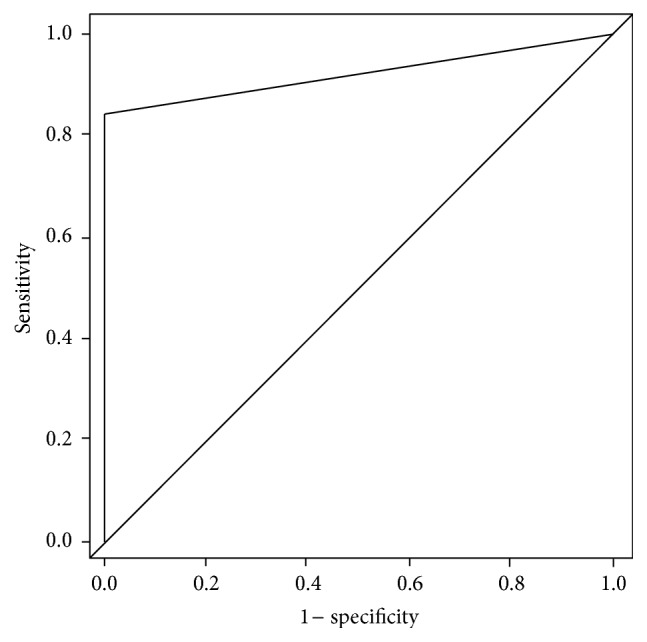
Receiver Operating Characteristic curve. ROC curve when using bronchoscopic to predict cancer and infection. Sensitivity 84.2% and specificity 100%.

**Figure 2:**
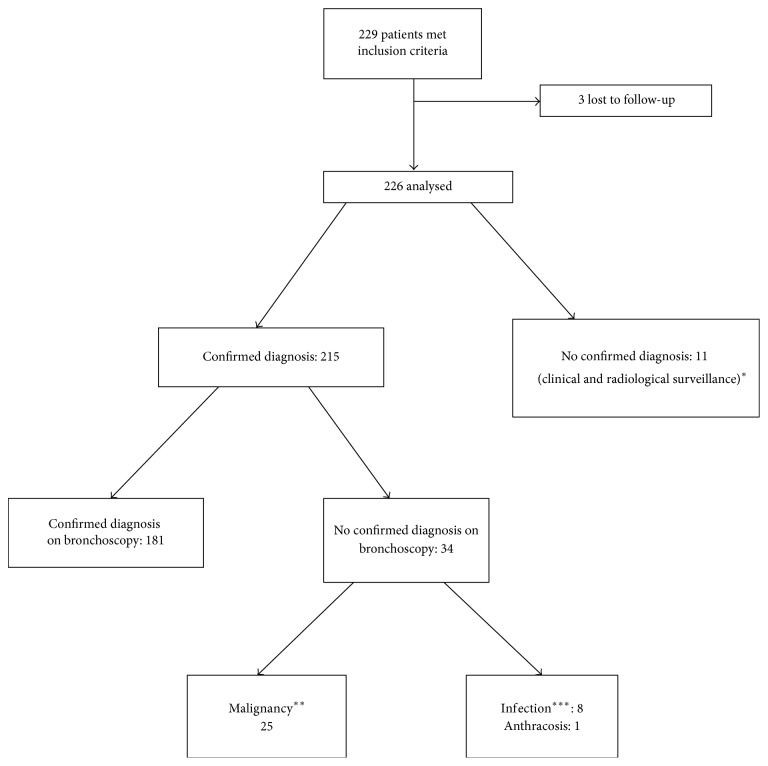
Diagnostic algorithm. 34 had nondiagnostic bronchoscopy and underwent repeated procedures with transthoracic CT-guided biopsy or surgical biopsy to obtain diagnosis. 11 out of 226 patients had nondiagnostic bronchoscopy results and were followed up with clinicoradiological surveillance. ^*∗*^Stable after 24 months of clinicoradiological surveillance. ^*∗∗*^Malignant disease: 22/25 were NSCLC of which 15 were adenocarcinoma, 1 was squamous cell carcinoma, and 6 were undifferentiated NSCLC. The other 3 were B cell lymphoma and metastatic breast carcinoma and the last patient was deceased but had a scan after TBLB with radiological evidence of metastatic cancer of unknown primary. ^*∗∗∗*^Infection: out of 8 cases, 5 were* Mycobacterium tuberculosis* complex, 1 nontuberculous* Mycobacterium*, and 2 fungal infections.

The diagnostic yield of bronchoscopy was 80.1% (181/226). The area under curve (AUC) of ROC (Figure 1) is 0.921 (0.896, 0.945) and showed that bronchoscopy had a sensitivity of 84.2% and specificity of 100% (Table 3 and Figure 1). The diagnostic yield of transbronchial lung biopsy (TBLB) alone excluding BAL results was 69.5% (157/226).

Lesions > 20 mm had a diagnostic yield of 84.9% compared to 63.0% in lesions ≤ 20 mm (*p* = 0.001). Cases with a positive CT-Bronchus sign had a trend towards a higher diagnostic yield (OR 1.745; 95% CI 0.853, 3.487; *p* = 0.1191) (Table 4). 142 cases (62.8%) had lesions that were both > 20 mm and had a positive CT-Bronchus sign. The diagnostic yield in this group was 83.8% (119/142) compared to 73.8% (62/84) who had neither or only one feature (*p* = 0.069). BAL cytology was available in 222 cases (98.2%). Only 5 cases had a negative diagnosis on forceps biopsy but were positive for malignancy on cytology from BAL. Overall, BAL accounted for 13.2% (24/181) of all positive bronchoscopic results not made by TBLB including all microbiological diagnoses.

Procedure related complications were documented in 48 patients (21.2%): 40 (17.7%) had mild to moderate bleeding (<50 mL) that responded either to bronchoscopic tamponade or to cold saline instillation, 6 (2.7%) had transient hypoxia (SpO_2_ < 85% only during the 2-hour postprocedure monitoring that required supplementary oxygen), and 2 (0.9%) had pneumothorax. Pneumothoraces resolved spontaneously and neither patient required chest tube insertion. Hypoxia was attributed to the sedation used and the comorbid conditions of the patient. None of the patients required escalation of care for complications. Logistic regression also showed that patients who had the upper lobe biopsied had a higher risk of developing complications [OR = 2.574, 95%  CI = (1.235, 5.801)] (Table 5).

## 4. Discussion

The diagnostic yield of conventional flexible bronchoscopy for peripheral lung lesions was higher in this study (80.1%) than expected based on historical data. Meta-analysis has showed a diagnostic sensitivity for TBLB to be 57% (21 studies) and for BAL/washing to be 43% (14 studies) [4]. If CT scanning showed a bronchus extending to the peripheral lesion, the yield of bronchoscopy was 60% compared to 25% if the CT-Bronchus sign was absent. The sensitivity for lesions < 20 mm was 34% compared to 63% if > 20 mm [4]. The American College of Chest Physicians Evidence-Based Clinical Practice Guidelines on the Diagnosis and Management of Lung Cancer recommends that in patients suspected of having lung cancer who have a peripheral lesion and who require tissue diagnosis before further management can be planned, CT-guided biopsy is a diagnostic option. The guidelines also recommend radial EBUS as an adjunct imaging modality based on a diagnostic sensitivity of 73% as derived from meta-analysis data [8]. Other data show that a positive CT scan Bronchus sign improves the overall yield of electromagnetic navigation bronchoscopy from 67% to 79% by multivariate analysis [9] hence leading to the recommendation that, in patients with peripheral lung lesions that are difficult to reach with conventional bronchoscopy, electromagnetic navigation guidance is recommended if the equipment and the expertise are available [4].

The data from our study involving procedures challenges many of these assertions. Diagnostic yield of 80% is possible with conventional flexible bronchoscopy for peripheral lung lesions suspected of being lung cancer. Patient selection is one reason for the high sensitivity. Targeted lesions were > 20 mm in size in 79.5% and the yield in this group was higher than in the ≤ 20 mm group. Patients with a positive CT-Bronchus sign accounted for 73.7% and this group had a higher trend towards a better diagnostic sensitivity. The proportion of patients who had at least 6 forceps biopsy specimens was 86.7%, the proportion who had multiple sampling modalities (BAL and TBLB) was 98.2% and the prevalence of malignancy was 78.7%. Therefore, following well-established criteria makes conventional bronchoscopy a reliable diagnostic modality in the workup of peripheral lung lesions. Moreover, our TBLB yield of 69.5% is comparable to the success of novel bronchoscopic procedures such as radial endobronchial ultrasound and electromagnetic navigation.

The results of the “nonoptimal” group may be even more compelling. The yield for ≤ 20 mm lesions was 63% and the rate for patients with a negative CT-Bronchus sign was 72.8%. One reason for these data may be enhanced planning with multiplanar CT thorax with fine cuts. Planning involves accurate localization of bronchopulmonary subsegments as well as determining a potential route of approach. The likely location of “fluoroscopically invisible” lesions can be determined using the coronal views and used as a guide when C-arm fluoroscopy was performed during TBLB. These features are already present in virtual bronchoscopy navigation and electromagnetic navigation. In addition, it is likely that using such planning and incorporating additional sampling modalities such as transbronchial needle aspiration may further improve the diagnostic yield in future [10]. If this hypothesis proves to be true, then there are implications in (1) retaining the role of conventional flexible bronchoscopy in the diagnosis of peripheral lung lesions and (2) avoiding unnecessary investment in expensive newer navigational bronchoscopic technologies with only marginal benefits. This data is all the more compelling given the recent publication of the AQuIRE registry where the diagnostic yield of electromagnetic navigation bronchoscopy (57.0%) and radial endobronchial ultrasound (38.5%) was lower than expected. In this multicenter registry, 53% of the lesions were >20 mm [10].

Several points of caution are noteworthy. The number of false negatives remains considerable at 34/215 (15.8%). Therefore, the recommendation that nondiagnostic bronchoscopic procedures be reevaluated by further investigations such as a CT-guided transthoracic biopsy or continued on radiological surveillance holds true. Although conventional flexible bronchoscopy is a safe procedure that can be done in an ambulatory setting under moderate sedation, complications in particular bleeding, hypoxia, and pneumothorax can occur. Endoscopists should anticipate such complications and be prepared to manage them, especially in cases with upper lobe lesions. The higher rate of complications in our study was attributed to the prospective nature of data collection.

Although this study shows data based on a large, prospective database in a “real-world” setting, it has several limitations. The favorable diagnostic yield of flexible bronchoscopy may be attributed to the superior planning offered by multiplanar CT thorax, but this can only be confirmed in a prospective randomized control trial. Despite strict institutional norms, bronchoscopy procedure and sampling modalities were also not fully standardized. In addition, a therapeutic (6 mm) bronchoscope was used for all cases, which is not optimal when navigating to peripheral lesions. Again this is reflective of “real-life” practice where a range of “thinner” bronchoscopes is not available to all pulmonologists. Data on the frequency of invisible nodules on fluoroscopy was also unavailable. However, our experience reflects how multiplanar CT may obviate the dependence on being able to visualize the lesions on fluoroscopy by using the coronal CT images as a guide. Finally, the yield of forceps biopsy and lavage was analyzed together instead of separately. The reason for this is that, even in a population with high prevalence (78.7%) of malignancy such as ours, lavage still yielded a microbiological or cytological diagnosis in 13.2%. This only reaffirms previous assertions that combined sampling adds to diagnostic yield [2, 3].

In conclusion, conventional flexible bronchoscopy with forceps biopsy and BAL/washing appears to retain a role in peripheral lung lesions that are suspicious for lung cancer. Appropriate patient selection (e.g., lesions > 20 mm and lesions with positive CT-Bronchus sign) and good endoscopic technique (≥6 forceps specimens and combined sampling modalities) are good guides to reliable bronchoscopic yields. Moreover, better radiology planning before the procedure may further enhance the diagnostic yield even in patients who do not meet such criteria.

## Figures and Tables

**Table 1:** Patient characteristics and procedure details. *n* (%).

Number of patients included in analysis	226

Male gender (%)	139 (61.5%)

Median age (range)	64 (30–90)

Median procedure duration in minutes (range)	25 (5–75)

Median number of biopsy specimens (Range)	8 (2–14)
Patients with at least 6 biopsy specimens (%)	196/226 (86.7%)
Diagnostic yield when ≥6 forceps specimens (%)	156/196 (79.6%), *p* = 0.633
Diagnostic yield when <6 forceps specimens (%)	25/30 (83.3%)

BAL	
Median volume instilled (range) in mL	60 (20–160)
Median percentage returns (range)	37.5 (13.0–83.0)

Sedation	
Median midazolam dose (range) in mg	2.5 (0–12.0)
Median fentanyl dose (range) in Mcg	50.0 (0–100.0)

Complications (%)	48/226 (21.2%)
Hypoxia (% of complications)	6/48 (12.5%)
Bleeding (% of complications)	40/48 (83.3%)
Pneumothorax (% of complications)	2/48 (4.2%)

Patients with lesions >20 mm (%)	179/225 (79.5%)
Diagnostic yield in lesions > 20 mm (%)	152/179 (84.9%), *p* = 0.001
Diagnostic yield in lesions ≤ 20 mm (%)	29/46 (63.0%)

CT-Bronchus sign present (%)	165/224 (73.7%)
Diagnostic yield in present CT-Bronchus sign (%)	136/165 (82.4%), *p* = 0.116
Diagnostic yield in absent CT-Bronchus sign (%)	43/59 (72.8%)

Overall positive diagnostic yield of bronchoscopic biopsy (%)	181/226 (80.1%)

**Table 2:** Positive diagnosis based on flexible bronchoscopy.

Diagnosis	*n* = 181 (%)
Adenocarcinoma	101 (55.8%)
Squamous cell carcinoma	14 (7.7%)
Metastases	12 (6.6%)
NSCC (unspecified)	26 (14.4%)
Tuberculosis	9 (5.0%)
Other infections^*∗*^	19 (10.5%)

Amongst patients with a positive bronchoscopy diagnosis, 84.5% were diagnosed with cancer and 15.5% were diagnosed with infections.

Other infections^*∗*^: 18 had bacterial infections and 1 had nontuberculous mycobacteria. All microbiological cultures were from bronchoalveolar lavage.

**Table 3:** Sensitivity and specificity of bronchoscopy.

	Confirmed diagnosis positive that is either malignancy or pulmonary infection	Confirmed diagnosis negative that is clinic-radiological pattern consistent with postinflammatory scar	Total
Bronchoscopy diagnosis positive	181	0	181
Bronchoscopy diagnosis negative	34	11	45

Total	215	11	226

Bronchoscopy had a sensitivity of 84.2% and specificity of 100%.

**Table 4:** Univariable logistic regression in predicting successful diagnosis of cancer or infection via bronchoscopy.

	OR (95% CI)	*p* value
Age	0.991 (0.962–1.021)	0.5590
Lesion size	1.104 (0.941–1.314)	0.2445
Number of biopsies	0.904 (0.763–1.070)	0.2375
Bronchus sign		
0 (absent)	Reference	
1 (present)	1.745 (0.853–3.487)	0.1191
Pleural Apposition		
0 (absent)	Reference	
1 (present)	1.429 (0.742–2.779)	0.2870
Location		
Lingular	Reference	
LLL	1.857 (0.403–7.968)	0.4070
LUL	2.939 (0.635–12.827)	0.1510
RLL	2.612 (0.562–11.450)	0.2020
RML	3.810 (0.682–23.818)	0.1290
RUL	2.143 (0.51–8.052)	0.2670

**Table 5:** Univariable logistic regression in predicting complications of bronchoscopy.

	OR (95% CI)	*p* value
Age	1.024 (0.995–1.054)	0.1148
Lesion size	1.103 (0.957–1.269)	0.1680
Number of biopsies	0.869 (0.731–1.025)	0.1030
Duration of bronchoscopy	0.985 (0.960–1.002)	0.2322
Midazolam dose	0.868 (0.700–1.051)	0.1692
Fentanyl dose	0.998 (0.988–1.008)	0.6959
Bronchus sign		
0 (absent)	Reference	
1 (present)	0.779 (0.399–1.565)	0.4709
Pleural Apposition		
0 (absent)	Reference	
1 (present)	1.290 (0.694–2.419)	0.422
Location		
Upper lobe biopsy		
0 (all other lobes)	Reference	
1 (upper lobes)	2.491 (1.308–4.919)	**0.0066**

## Abbreviations

CT: Computed tomography
BAL: Bronchoalveolar lavage
TBLB: Transbronchial lung biopsy.

## Additional Points

Previous data show that conventional bronchoscopy in patients with peripheral lung lesions has limited diagnostic yield and is dependent on lesion size. Our study suggests that, with careful patient selection and preprocedure planning using multiplanar computed tomography, conventional bronchoscopy retains its role in the diagnosis of these lesions.
